# Bioactivities of β-mangostin and its new glycoside derivatives synthesized by enzymatic reactions

**DOI:** 10.1098/rsos.230676

**Published:** 2023-08-16

**Authors:** Tuoi Thi Le, Nguyen Thu Trang, Van Thuy Thi Pham, Dang Ngoc Quang, Le Thi Phuong Hoa

**Affiliations:** ^1^ Faculty of Biology, Hanoi National University of Education, 136 Xuan Thuy, Cau Giay, Hanoi 100000, Vietnam; ^2^ Faculty of Chemistry, Hanoi National University of Education, 136 Xuan Thuy, Cau Giay, Hanoi 100000, Vietnam; ^3^ Institute of Microbiology and Biotechnology, Vietnam National University, 144 Xuan Thuy, Cau Giay, Hanoi 100000, Vietnam

**Keywords:** acetylcholinesterase, β-mangostin, glycosylation

## Abstract

Beta-mangostin is a xanthone commonly found in the genus *Garcinia*. Unlike α-mangostin, to date, there have only been a few studies on the biological activity and derivatization of β-mangostin. In this study, two novel glycosylated derivatives of β-mangostin were successfully synthesized via a one-pot enzymatic reaction. These derivatives were characterized as β-mangostin 6-O-β-d-glucopyranoside and β-mangostin 6-O-β-d-2-deoxyglucopyranoside by TOF ESI/MS and ^1^H and ^13^C NMR analyses. Beta-mangostin showed cytotoxicity against KB, MCF7, A549 and HepG2 cancer cell lines, with IC_50_ values ranging from 15.42 to 21.13 µM. The acetylcholinesterase and α-glucosidase inhibitory activities of β-mangostin were determined with IC_50_ values of 2.17 and 27.61 µM, respectively. A strong anti-microbial activity of β-mangostin against Gram-positive strains (*Bacillus subtilis*, *Lactobacillus fermentum* and *Staphylococcus aureus*) was observed, with IC_50_ values of 0.16, 0.18 and 1.24 µg ml^−1^, respectively. Beta-mangostin showed weaker activity against Gram-negative strains (*Salmonella enterica*, *Escherichia coli* and *Pseudomonas aeruginosa*) as well as *Candida albicans* fungus, with IC_50_ and MIC values greater than the tested concentration (greater than 32 µg ml^−1^). The new derivatives of β-mangostin showed weaker activities than those of β-mangostin, demonstrating the important role of the hydroxyl group at C-6 of β-mangostin in its bioactivity.

## 1. Introduction

Beta-mangostin is a common xanthone found in the genus *Garcinia*, which includes *G. mangostana*, *G. oliveri* and *G. cowa*. Unlike α-mangostin, so far, there have been few studies on the biological activity of β-mangostin and its new derivatization. There are a few reports on the anti-cancer activity of β-mangostin, such as growth inhibition in MCF-7 breast cancer cells [1], murine leukaemia cells (WEHI-3) [2], inhibition of migration and invasion of human hepatocellular carcinoma (HCC) cells [3] and oral squamous cell carcinoma [4]. To the best of our knowledge, there are no reports on the α-glucosidase-inhibitory activity of β-mangostin. To date, there has been one report on new derivatives of β-mangostin and their anti-inflammatory activity [5].

Similar to other xanthone compounds, β-mangostin has poor water solubility, which limits its bioavailability. Therefore, an enzymatic glycosylation reaction can alter the molecular structure, improve the solubility and alter the bioactivity of β-mangostin. Glycosylation is a natural reaction catalysed by glycosyltransferases (GTs), which form a glycosidic bond between the hydroxyl group of the aglycone and the sugar moiety using NDP-sugar (e.g. UDP-glucose). In this study, GT-YjiC, which has been reported to have high flexibility, contained small aglycones, such as flavonoids [6,7], macrolides [8] and xanthones (α-mangostin) [9] as well as NDP-sugar donors such as UDP-glucose and TDP-2-deoxyglucose [9]. An increase in the biological activity of glycosylated derivatives has previously been demonstrated. The antibacterial activity of α-mangostin against Gram-positive bacteria was increased by 1.5–2.5 times when α-mangostin was glycosylated with a glucose moiety at C-3 [9]. The anti-tumour activity of curcumin was enhanced by 4′-O-β-glucoside and 4′-O-β-2-deoxyglucoside derivatives [10].

In this study, two new glycosylation derivatives of β-mangostin synthesized by enzymatic reactions were reported, together with the cytotoxic activity in various cancer cell lines including epidermal carcinoma (KB), breast cancer cell line (MCF-7), hepatocellular carcinoma (HepG2), pulmonary carcinoma (A549), and anti-microbial as well as acetylcholinesterase (AChE) and α-glucosidase inhibitory activities of β-mangostin and its novel glycosyl derivatives.

## 2. Material and methods

### 2.1. Materials

Beta-mangostin and α-mangostin were purchased from Chengdu Biopurify Phytochemicals, Ltd. Recombinant *Escherichia coli* (BL21 (DE3)) strains were kindly provided by Prof. Jae Kyung Sohng, Sun Moon University, Korea. The cell lines and microorganisms were derived from the American Type Culture Collection (ATCC). α-Glucosidase (CAS no. 900-42-7, Sigma) was from *Saccharomyces cerevisiae.* All chemicals were of analytical grade and available.

### 2.2. Methods

#### 2.2.1. Enzymatic synthesis of β-mangostin derivaties

Recombinant proteins (UMK-uridine monophosphate kinase, ACK-acetate kinase, NAGK-*N*-acetyl-d-glucosamine kinase, GalU-glucose-1-phosphate uridylyltransferase, PMM-phosphomannomutase and YjiC-glycosyltransferase) were used and reaction conditions for β-mangostin glycosylation were according to the procedure described by Le *et al*. [9]. Briefly, for each reaction, 2 mM β-mangostin was used as initial concentration in combination with 2 mM UMP, 200 mM acetyl phosphate, 1 mM ATP, 20 mM MgCl_2_ and 100 mM Tris buffer. For UDP-glucose reaction system, 50 mM glucose-1-phosphate and 50 µg ml^−1^ of each recombinant protein (UMK, ACK, GalU and YjiC) were added while UDP-2-deoxyglucose reaction system, 50 mM of 2-deoxyglucose was used with six recombinant proteins (N-AGK, PMM, YjiC, GalU, ACK and UMK) [9].

The reactions were kept at 37°C under shaking conditions (100 rpm) for 5 h and tested by thin-layer chromatography (ethyl acetate : methanol : water = 8 : 1.5 : 0.5). Depending on the yield of each system, β-mangostin substrate was added to achieve maximum conversion. The final products were analysed by high-performance liquid chromatography (HPLC).

#### 2.2.2. Analysis of β-mangostin glycosylated products

HPLC analysis was performed on a Thermo Fisher UltiMate 3000 HPLC (Thermo Scientific) with an Extrasil AQ-C18 column (5 µm × 4.6 mm × 250 mm). The HPLC conditions were according to Le *et al*. [9].

Preparative HPLC was developed based on the HPLC method using a Combi Flash NEXTGEN 300+ (TELEDYNE ISCO, USA). A reversed-phase column (13 g, C18 RediSepRf) was used with UV absorbances of 310 nm. The binary mobile phases were solvent A (HPLC-grade water) and solvent B (100% acetonitrile, CH_3_CN). The concentrations of acetonitrile used were: 20**–**100% (0**–**18 min) and 100% (18**–**25 min) and flow rate was 13 ml min^−1^.

The purified products were dried, lyophilized, dissolved in DMSO-*d*_6_ and analysed using ^1^H and ^13^C NMR (nuclear magnetic resonance) spectroscopy (Bruker 600 MHz spectrometer) and ESI/MS (LC-MSD-Trap-SL).

#### 2.2.3. Cytotoxicity assay

The cytotoxic activities against the cancer cell lines KB epidermal carcinoma, A549 lung cancer, HepG2 liver cancer and MCF-7 breast cancer, as well as the healthy cell line HEK293 embryonic kidney cells, were determined using the 3-(4,5-dimethylthiazol-2-yl)-2,5-diphenyltetrazolium bromide (MTT) assay [11,12]. Briefly, cell lines were grown into log phase at 37°C, 5% CO_2_ in DMEM-Dulbecco's modified Eagle's medium and 10% fetal bovine serum. A concentration of 1 × 10^4^–3 × 10^4^ cells ml^−1^ was prepared depending on the cell line used for the experiment by adding 10 µl of the sample and 190 µl of the cell suspension, followed by incubation under standard conditions. After 72 h of culture, 10 µl of MTT (5 mg ml^−1^) was added and incubated for 4 h. Dimethyl sulfoxide (100 µl) was added to dissolve formazan crystals and the density was measured at 540 nm using a BioTek spectrophotometer. The inhibition of cell growth was expressed as IC_50_ (the half-maximal inhibitory concentration) value. Ellipticine was used as reference.

#### 2.2.4. Acetylcholinesterase inhibitory activity assay

For the assay, acetylthiocholine iodide (ATCI) was used as a substrate for the reaction and 5,5′-dithiobis-(2-nitrobenzoic acid) (DTNB) was used to measure AChE activity [13,14]. The reaction (200 µl) on a 96-well plate was performed by mixing Tris–Cl buffer (pH 8.0), tested sample in a twofold serial dilution, AChE (0.25 IU ml^−1^) and incubating at 25°C, 15 min before adding 2.4 mM DTNB and 2.4 mM ATCI. The reaction mixture was mixed well and further incubated for 15 min and was measured at 412 nm (A). The ability of the tested samples to inhibit AChE activity was determined as the IC_50_ (μM), the concentration that reduced 50% of AChE activity with reference to the control (donepezil).

#### 2.2.5. Alpha-glucosidase inhibitory assay

The α-glucosidase inhibitory activity assay [15,16] was conducted using a reaction mixture containing phosphate buffer (100 mM; pH 6.8), *p*-nitrophenyl-α-d-glucopyranoside (pNPG) (2.5 mM) and samples at various concentrations. The reactions were initiated by adding α-glucosidase (0.2 U ml^−1^), incubated at 37°C for 30 min and quenched by adding Na_2_CO_3_ (50 mM). The absorbance of the reaction was determined using a BioTek instrument at a wavelength of 410 nm (A). The inhibition of α-glucosidase activity was determined as IC_50_ (μM) with acarbose was a positive control.

#### 2.2.6. Anti-microbial activity assay

Bacterial and fungal strains (*Bacillus subtilis*, *Staphylococcus aureus*, *Lactobacillus fermentum*, *Escherichia coli*, *Pseudomonas aeruginosa*, *Salmonella enterica* and *Candida albicans*) were activated on TSA (Tryptic Soy Agar) and SA (Sabouraud–4% dextrose agar) media, respectively. Before the experiment, the microbial strains were grown in the corresponding liquid media at a concentration of approximately 5 **×** 10^5^ CFU ml^−1^ for bacteria and 1 **×** 10^3^ CFU ml^−1^ for fungi. The microbial suspension (190 µl) was mixed with 10 µl of the test sample, which was serially diluted twofold, and the microplate was incubated at 37°C for 16–24 h. The MIC value was determined for the well with the lowest sample concentration that completely inhibited microbial growth [17,18].

## 3. Results and discussion

### 3.1. Glycosylation of β-mangostin by enzymatic systems

**Figure 1.**
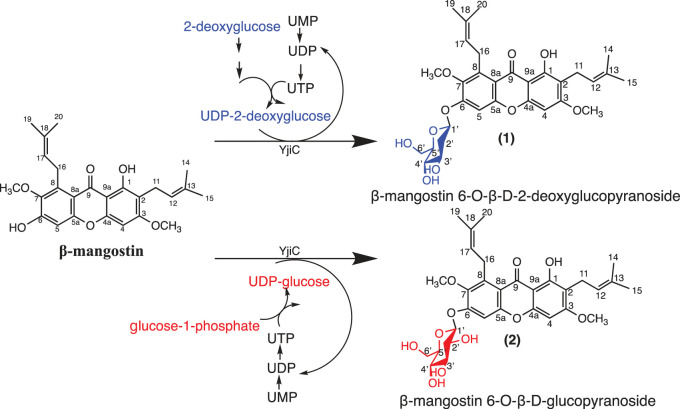
Schematic diagram for the synthesis of new β-mangostin glycosides.

**Figure 2.**
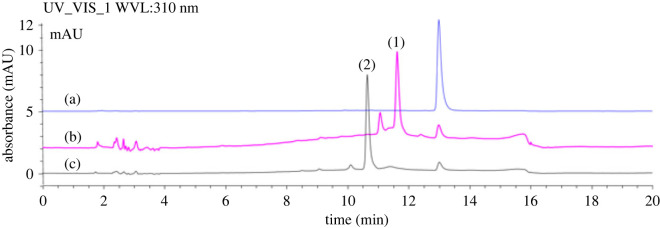
HPLC analysis of (*a*) β-mangostin standard; (*b*) products of the reaction using UDP-2-deoxyglucose system with the new product (**1**); (*c*) products of the reaction using UDP-glucose system with the new product (**2**).

Two *in vitro* systems for synthesizing UDP-glucose and UDP-2-deoxyglucose, in combination with a glycosyltransferase (YjiC) from *Bacillus licheniformis* were used to synthesize new glycoside derivatives of β-mangostin (figure 1). With the advantages of the two systems that use recycled UDP and ATP, along with the flexibility of YjiC, these systems demonstrated the efficient synthesis of new derivatives of β-mangostin with new peaks (1) and (2) (figure 2). The conversion was almost complete after 5 h of reaction (approx. 94%) with 2 mM and 3 mM of β-mangostin as substrate for the reaction system with UDP-glucose and UDP-2-deoxyglucose, respectively (figure 2).

Compounds (1) and (2) were purified by preparative HPLC and analysed by mass spectrometry and NMR. The TOF ESI/MS of compound (1) showed a peak of [M + H]^+^ ions at *m/z* 571.2457, which confirmed the glycoside product of β-mangostin conjugated with the 2-deoxyglucose residue. Similarly, the ion peak [M + H]^+^ of compound (2) was at 587.0, corresponding to the formula C_31_H_38_O_11_, a β-mangostin glucoside. The structures of these new products were further analysed using ^1^H and ^13^C NMR spectroscopy.

The ^1^H NMR spectra of compounds (1) and (2) showed an anomeric proton signal at *δ* 4.62 (d, 2H), confirming the presence of a sugar residue (table 1). The position for the attachment of sugar residue of both compounds was C-6 since the proton signals at position C-5 (*δ* 7.15 and 7.18 ppm for compounds (1) and (2), respectively) were shifted to those reported in the literature for β-mangostin [19]. The sugar moiety of compound (1) was 2-deoxyglucose as the signals of protons of C-2′ appeared at *δ* 2.27 and 1.35 ppm (table 1) [20]. This was also a different signal from the proton signal of the glucose moiety of compound (2). Therefore, compound (1) was confirmed to be β-mangostin 6-O-β-d-2-deoxyglucopyranoside (β-Man2DG) and compound (2) was β-mangostin 6-O-β-d-glucopyranoside (β-ManGlc).

**Table 1.** ^1^H and ^13^C NMR data for the β-mangostin glycosides.

position	compound (1): β-mangostin 6-O-β-d-2-deoxyglucopyranoside		compound (2): β-mangostin 6-O-β-d-glucopyranoside	
	**^1^H(*δ*)**	**^13^C(*δ*)**	**^1^H(*δ*)**	**^13^C(*δ*)**
1	—	158.72	—	158.71
2	—	110.54	—	110.50
3	—	163.46	—	163.43
4	6.64 (s, 1H)	89.52	6.64 (s, 1H)	89.52
4a	—	155.36	—	155.93
5	7.15 (s, 1H)	102.02	7.18 (s, 1H)	101.82
5a	—	154.34	—	154.33
6	—	154.83	—	154.83
7	—	143.86	—	143.99
8	—	136.21	—	136.14
8a	—	111.81	—	111.77
9	—	181.59	—	181.59
9a	—	102.91	—	102.91
11	3.31	20.86	3.31	20.85
12	5.05 (m, 1H)	123.22	5.09 (m, 1H)	123.23
13	—	130.64	—	130.62
14	1.78 (s, 3H)	17.58	1.79 (s, 3H)	17.57
15	1.62 (s, 3H)	25.42	1.62 (s, 3H)	25.40
16	4.05 (d, 2H)	25.59	4.07	25.58
17	5.14 (m, 1H)	121.99	5.14 (m, 1H)	121.98
18	—	130.82	—	130.82
19	1.73 (s, 3H)	17.94	1.62 (s, 3H)	17.93
20	1.62 (s, 3H)	25.49	1.73 (s, 3H)	25.48
1-OH	13,46 (s, 1H)	—	13,49 (s, 1H)	—
3-OCH_3_	3.92 (s, 3H)	56.31	3.92 (s, 3H)	56.31
6-OH	—	—	—	—
7- OCH_3_	3.73 (s, 3H)	60.85	3.78 (s, 3H)	60.67
1′	4.62 (d, 2H)	96.75	4.62 (d, 2H)	99.93
2′	2.27, 1.35	34.37	2.38	73.17
3′	3.1	77.56	3.2	76.55
4′	2.68	70.06	2.63	69.6
5′	3.2	78.64	3.2	77.25
6′	3.4, 3.5	60.48	3.4, 3.7	60.59

### 3.2. Bioactivities of β-mangostin and its glycoside derivatives

#### 3.2.1. Cytotoxic activity

The cytotoxicity of β-mangostin and its glycoside derivatives, β-Man2DG (1) and β-ManGlc (2), was investigated using an MTT assay [11,12]. Beta-mangostin showed toxicity in all tested cell lines, with IC_50_ values ranging from 15.42 to 21.13 μM (table 2). Meanwhile, the glycoside derivatives had higher IC_50_ values, higher than 50 μM in the case of β-ManGlc and a range of 59.24**–**92.3 μM in the case of β-Man2DG for all tested cell lines.

**Table 2.** Cytotoxicity of β-mangostin and its glycoside derivatives on various cell lines. Values are expressed as mean ± s.d. for three replicates.

cell line	IC_50_ (μM)				
	α-mangostin	β-mangostin	β-Man2DG (**1**)	β-ManGlc (**2**)	ellipticine
KB	15.4 ± 1.26	21.13 ± 9.9	59.24 ± 3.87	>50	1.3 ± 0.08
MCF-7	81.9 ± 6.09	18.04 ± 8.1	78.96 ± 7.9	>50	1.67 ± 0.12
A549	22.7 ± 1.21	15.42 ± 5.1	85.4 ± 6.5	>50	1.26 ± 0.08
HepG2	0.24 ± 0.21	18.86 ± 7.6	92.3 ± 2.3	>50	1.67 ± 0.16
HEK293	25.4 ± 1.95	15.6 ± 6.5	84.12 ± 6.3	>50	4.39 ± 0.56

The cytotoxic activity of β-mangostin on the MCF-7 cell line with an IC_50_ value of 18.04 μM for 72 h treatment was similar to that reported by Syam *et al*. [1], with an IC_50_ of 16.5 μM for 48 h and 26 μM for 24 h treatment [1]. According to Syam *et al*. [1], β-mangostin induces p53-dependent G2/M cell cycle arrest and apoptosis via reactive oxygen species (ROS)-mediated mitochondrial pathway and NF-kB suppression [1]. Interestingly, the cytotoxic activity of β-mangostin against MCF-7 cells was 4.45 times stronger than that of α-mangostin (IC_50_ = 81.9 μM) (table 2). According to previous studies on MCF-7 cells, α-mangostin induces apoptosis, inhibits metastasis [21] and activates MOAP-1 tumour suppressor and mitochondrial signalling [22].

The cytotoxic activity of β-mangostin has also been reported in other cell lines, and its IC_50_ value varies widely. In HeLa cervical cancer cells, the IC_50_ value was 27.2 µM [23]. On glioma cells, β-mangostin inhibited cell survival at IC_50_ values of 4.8, 5.8 and 10.3 µM for C6, U251 and T89G cells, respectively [24]. Several mechanisms of β-mangostin activity have also been revealed, such as inhibitory effects on DNA polymerases and topoisomerases, cell cycle arrest at the G1 and S phases, apoptosis in C6 cells, and mitochondrial function impairment and suppression of the PI3K, AKT and mTOR signalling pathways [24].

In this study, the cytotoxic activity of the β-mangostin glycosyl derivatives was lower than that of β-mangostin. However, further research on the mechanisms of apoptosis, metastasis and related signalling pathways is required to clarify the role of these new derivatives. It has been previously reported that β-mangostin did not cause cytotoxicity or cell cycle suppression in hepatocellular carcinoma cell lines (Huh-7, SK-Hep-1, HA22T/VGH cell lines) [3,24] or HeLa and SiHa cells [24,25], but inhibited cell migration and invasion. According to Kim *et al*. [26], two deoxyglucoside derivatives of α-mangostin, Man-3DG (α-mangostin 3-O-β-d-2-deoxyglucopyranoside) and Man-6DG (α-mangostin 6-O-β-d-2-deoxyglucopyranoside), suppressed the growth and migration of hepatocellular carcinoma cell lines (Huh-7, Hep3B, HepG2) [26]. In addition, by controlling mitochondrial apoptosis-related proteins in Hep3B cells, these compounds induce apoptosis. Man-3DG and Man-6DG suppressed angiogenesis induced by Hep3B cells *in vitro* by downregulating hypoxia-inducible factor-1 and endothelial vascular growth factor [26].

Alpha-mangostin and β-mangostin exhibited different toxicities in the five tested cell lines. Beta-mangostin exhibited a fairly stable effect with IC_50_ values ranging from 15.42 to 21.13 μM, while α-mangostin exhibited IC_50_ values with broader range, from 0.24 μM to 81.9 μM depending on the cell line (table 2). In terms of molecular structure, these molecules differ only at the C-3 position, with C3-OH in α-mangostin and C3-OCH_3_ in β-mangostin, suggesting that C3-OH may interact with different signalling molecules in different cell lines. In addition, the C6-glycoside (2-deoxyglucose) of α-mangostin had similar toxicity to α-mangostin [26], whereas the 2-deoxyglucoside of β-mangostin showed 4.8-fold lower activity than that of alycone. Therefore, further studies on the mechanism of these derivatives need to be conducted to better evaluate the relationship between their structure and cytotoxic activity to develop anti-cancer drugs.

#### 3.2.2. Inhibition of acetylcholinesterase activity

AChE plays the main role in hydrolysis of acetylcholine (90%) along with butyrylcholinesterase (BuChE) to regulate cholinergic neurotransmission [27–29]. AChE inhibitors can delay the progress of mental illness and reduce neuropsychiatric symptoms, thus providing a rational therapeutic approach to Alzheimer's disease treatment.

The results in table 3 show that β-mangostin had a significant inhibitory effect on AChE, whereas glycoside derivatives (1) and (2) both had weaker effects. The AChE inhibitory activity of β-mangostin was similar to that of α-mangostin (2.14 μM), as reported by Khau *et al*. [30].

**Table 3.** Inhibition of AChE of β-mangostin and its glycoside derivatives**.** Values are expressed as mean ± s.d. for three replicates.

enzyme	IC_50_ value (μM)			
	β-mangostin	β-Man2DG (**1**)	β-ManGlc (**2**)	donepezil
AChE	2.17 ± 0.18	>124	>50	0.047 ± 0.008

According to Chi *et al*. [31], a series of α-mangostin derivatives was designed, synthesized and evaluated for their inhibitory activity against AChE and BuChE. Among the derivatives, the most potent inhibitor of AChE was compound (16) (4-bromo-1,3,6-trihydroxy-2,8-diisopentyl-7-methoxy-9*H*-xanthen-9-one) while compound (5) (2-(2,3-dihydroxy-3-methylbutyl)-1,3,6-trihydroxy-8-isopentyl-7-methoxy-9H-xanthen-9-one) was the most potent inhibitor of BuChE with IC_50_ values of 5.26 μM and 7.55 μM, respectively [31]. Similarly, a recent report by Yang *et al*. [32] showed that among four xanthone derivatives that were synthesized, compound 3-(2-(pyrrolidinyl)ethoxy)-1- hydroxy-9H-xanthen-9-one showed the highest AChE inhibitory activity and high specificity for AChE (IC_50_ = 2.403 µM) in comparison with BuChE (IC_50_ = 31.221 µM) [32].

Currently, four drugs are used for treatment of Alzheimer's disease: donepezil, rivastigmine, galantamine and the glutamate antagonist memantine. Galantamine, an alkaloid extracted from the Amaryllidaceae family, is a naturally occurring AChE inhibitor. The advantage of natural AChE inhibitor substances is that in addition to anti-AChE activity, there are other beneficial activities, such as antioxidant activity [33–35]. Several recent studies have been conducted to identify and isolate natural molecules applicable to the design and development of new anti-Alzheimer's disease drugs [36,37]. Our study also found that the natural compound β-mangostin has high potential for AChE inhibition (IC_50_ value of 2.17 µM) in comparison to synthesized derivatives. Similar natural xanthone compounds including α-mangostin and γ-mangostin had strong inhibitory activity against AChE with IC_50_ values of 2.14 µM and 1.31 µM, respectively [30]. In addition to the causes of Alzheimer's disease by impaired neurotransmission, some others have also been studied and become the target in the treatment of Alzheimer's disease such as: accumulation of pathological proteins, e.g. amyloid-β protein, tau tangles and neuroinflammation activation, and increased oxidative stress [38–41].

#### 3.2.3. Inhibition of α-glucosidase activity

Alpha-glucosidase is an important enzyme that affects the blood glucose levels. These enzyme inhibitors slow the release of d-glucose and reduced blood sugar levels. Therefore, one strategy for the treatment of diabetes is inhibition of α-glucosidase.

From the results in table 4, β-mangostin showed strong inhibitory activity against α-glucosidase (IC_50_ = 27.62 µM) when compared with the positive control acarbose (IC_50_ = 208.62 µM), with an inhibitory potency ratio of 7.55. All glycoside derivatives seemed to lose this activity, with IC_50_ values higher than the tested concentrations (greater than 124 µM for β-Man2DG and greater than 50 µM for β-ManGlc). This result is similar to that of α-mangostin, with α-glucosidase inhibitory activity at an IC_50_ of 31.1 µM, while synthetic compounds (CS1-CS4, derivatives of xanthenone substituted at position 1 with various side chains) lost this activity [42].

**Table 4.** Inhibition of α-glucosidase of β-mangostin and its glycoside derivatives**.** Values are expressed as mean ± s.d. for three replicates.

enzyme	IC_50_ value (μM)			
	β-mangostin	β-Man2DG (**1**)	β-ManGlc (**2**)	acarbose
α-glucosidase	27.61 ± 4.6	>124	>50	208.62 ± 4.7

The structural relationship and α-glucosidase inhibitory activity were also determined by analysing the IC_50_ values of the investigated substances, such as synthetic hydroxylxanthones [43]. The results showed that all eight hydroxylated derivatives of xanthone had 21 times higher α-glucosidase inhibitory activity than that of the parent xanthone, and there was a correlation between enzyme inhibitory activity and the number of hydroxyl groups, such as 1,3,7-trihydroxyxanthone, which had 12-fold stronger activity than 1-hydroxyxanthone [43]. Similarly, the α-glucosidase inhibitory activities of α-mangostin, β-mangostin and γ-mangostin were investigated in a study by Ryu *et al*. [44], showing a close relationship between the number of hydroxyl groups in the A and B rings and inhibitory potential in the order γ-mangostin (IC_50_ = 1.5 μM) > α-mangostin (IC_50_ = 5.0 μM) > β-mangostin (IC_50_ = 14.4 μM) [44]. This may also explain why glycosylated derivatives of β-mangostin had lower α-glucosidase inhibitory activity when the C6-OH group was glycosylated.

#### 3.2.4. Anti-microbial activity

The results in table 5 show that β-mangostin was able to inhibit *B. subtilis* and *L. fermentum* with the same MIC values of 0.5 μg ml^−1^ and IC_50_ values of 0.16 μg ml^−1^ and 0.18 μg ml^−1^, respectively. For the remaining strains, β-mangostin and its derivatives (1) and (2) exhibited weak inhibitory activity, with MIC values higher than the tested concentrations.

**Table 5.** IC_50_ and MIC values of β-mangostin and its glycoside derivatives against microorganisms. Values are expressed as mean ± s.d. for three replicates.

	*S. aureus*	*B. subtilis*	*L. fermentum*	*S. enterica*	*E. coli*	*P. aeruginosa*	*C. albicans*
IC_50_ (μg ml^−1^)							
*β*-mangostin	1.24 ± 0.05	0.16 ± 0.01	0.18 ± 0.01	>32	>32	>32	>32
*β*-Man2DG (1)	>71	>71	68.2 ± 1.98	>71	>71	>71	>71
*β*-ManGlc (2)	>29	>29	>29	>29	>29	>29	>29
ampicillin	0.02 ± 0.005	3.62 ± 0.15	1.03 ± 0.07				
cefotaxime				0.43 ± 0.05	0.007 ± 0.002	4.34 ± 0.15	
nystatin							1.32 ± 0.05
MIC (μg ml^−1^)							
*β*-mangostin	>32	0.5	0.5	>32	>32	>32	>32
*β-*Man2DG (1)	>71	>71	>71	>71	>71	>71	>71
*β-*ManGlc (2)	>29	>29	>29	>29	>29	>29	>29
ampicillin	0.125 ± 0.0	32 ± 0.0	32 ± 0.0				
cefotaxime				32 ± 0.0	0.5 ± 0.0	8 ± 0.0	
nystatin							8 ± 0.0

In a previous report on the antibacterial activity of xanthone against *S. aureus* (MRSA; methicillin-resistant strain) and *Enterococcus* (VRE; vancomycin-resistant strain), β-mangostin showed no antibacterial activity (MIC > 200 μg ml^−1^), whereas α- and γ-mangostin exhibited the highest activities among the eight xanthones studied (MICs ranging from 3.13 to 6.25 μg ml^−1^) [45]. Based on those results, it was suggested that the C3-OH, C6-OH and C2-prenyl chain were essential for antibacterial activities of α-mangostin and γ-mangostin [45]. Therefore, further research on the antibacterial mechanism of xanthones and their derivatives is required to understand the role of these functional groups.

## 4. Conclusion

The natural compound β-mangostin has potential activities associated with cancer cell toxicity, inhibition of AChE and α-glucosidase, and Gram-positive antibacterial activity. Two new derivatives of β-mangostin, β-mangostin 6-O-β-d-2-deoxyglucopyranoside (1) and β-mangostin 6-O-β-d-glucopyranoside (2), were synthesized by enzymatic methods. Although the biological activities of these derivatives have been reduced compared to those of β-mangostin, further research is required to clarify the role of the functional groups of these derivatives in the mechanisms of anti-cancer, antibacterial, antidiabetic and anti-Alzheimer effects.

## Data Availability

The data are provided in electronic supplementary material [46].
